# Bronchial airway gene expression in smokers with lung or head and neck cancer

**DOI:** 10.1002/cam4.190

**Published:** 2014-02-04

**Authors:** Eric Van Dyck, Petr V Nazarov, Arnaud Muller, Nathalie Nicot, Manon Bosseler, Sandrine Pierson, Kris Van Moer, Valérie Palissot, Céline Mascaux, Ulrich Knolle, Vincent Ninane, Romain Nati, Roy M Bremnes, Laurent Vallar, Guy Berchem, Marc Schlesser

**Affiliations:** 1 Département d'Oncologie, CRP-Santé du LuxembourgLuxembourg; 2 Unité de Recherche en Génomique, CRP-Santé du LuxembourgLuxembourg; 3 Princess Margaret Hospital and the University of TorontoOntario, Canada; 4 Laboratoire National de SantéLuxembourg; 5 Service de Pneumologie, CHU Saint-PierreBrussels, Belgium; 6 Service de Pneumologie, CHLLuxembourg; 7 Institute of Clinical Medicine, University of TromsøTromsø, Norway; 8 Service d'Oncologie, CHLLuxembourg

**Keywords:** Bronchial biopsy, cigarette smoking, gene expression microarrays, head and neck cancer, non-small cell lung cancer

## Abstract

Cigarette smoking is the major cause of cancers of the respiratory tract, including non-small cell lung cancer (NSCLC) and head and neck cancer (HNC). In order to better understand carcinogenesis of the lung and upper airways, we have compared the gene expression profiles of tumor-distant, histologically normal bronchial biopsy specimens obtained from current smokers with NSCLC or HNC (SC, considered as a single group), as well as nonsmokers (NS) and smokers without cancer (SNC). RNA from a total of 97 biopsies was used for gene expression profiling (Affymetrix HG-U133 Plus 2.0 array). Differentially expressed genes were used to compare NS, SNC, and SC, and functional analysis was carried out using Ingenuity Pathway Analysis (IPA). Smoking-related cancer of the respiratory tract was found to affect the expression of genes encoding xenobiotic biotransformation proteins, as well as proteins associated with crucial inflammation/immunity pathways and other processes that protect the airway from the chemicals in cigarette smoke or contribute to carcinogenesis. Finally, we used the prediction analysis for microarray (PAM) method to identify gene signatures of cigarette smoking and cancer, and uncovered a 15-gene signature that distinguished between SNC and SC with an accuracy of 83%. Thus, gene profiling of histologically normal bronchial biopsy specimens provided insight into cigarette-induced carcinogenesis of the respiratory tract and gene signatures of cancer in smokers.

## Introduction

Cigarette smoking accounts for 85–90% of lung cancer 1 and is a major risk factor for head and neck (HN) cancers 2. The high mortality rate of lung cancer (5-year survival rate of only 8–15% 3) and, to a lesser extent, that of head and neck cancer (HNC) 4, is mainly a consequence of late diagnosis and lack of efficient treatment for advanced-stage disease. A better understanding of the early stages of carcinogenesis is crucial to improve disease diagnostic tools and treatments, and should also help explain why only a fraction of all smokers develop cancer of the respiratory tract.

Cigarette smoking causes oxidative stress 5, drives inflammation 6, and leads to the accumulation of genetic and epigenetic abnormalities 7,8, and altered gene expression 9 throughout the respiratory tract. This molecular field of injury 7 reflects both damages induced by the chemicals in cigarette smoke, and the host response to these chemicals. Large-scale gene-expression profiling analyses have been undertaken to explore the field of smoking-induced injury, in relation to lung cancer (ref 10 and references therein) and HNC 11. These studies have revealed a number of differentially regulated genes and molecular pathways associated with disease pathogenesis and identified potential lung cancer biomarkers. For instance, an 80-gene biomarker distinguishing smokers with or without lung cancer with an accuracy of 83% was identified through profiling of cytologically normal, epithelial cell brushings of the airway 12. Importantly, smoking-related gene expression changes detected in the lower airways were reflected in cytological smears of nasal and buccal epithelium 13,14.

Tumor-distant, histologically normal bronchial biopsies have hitherto not been considered in gene expression studies of lung or HN carcinogenesis 9,12,15–17. However, such biopsies may constitute an important temporal gate to understand carcinogenesis, providing information not only from airway epithelial cells but also sub-epithelial cells and inflammatory/immune cells implicated in protection against cancer. In addition, unlike tumor-adjacent tissues from resected specimens, gene expression in tumor-distant biopsy specimens is less likely to be influenced by the tumor itself.

In this study, we have carried out gene expression profiling of histologically normal bronchial biopsy specimens from healthy individuals as well as current smokers with or without non-small cell lung cancer (NSCLC) or HNC, in order to identify differentially expressed genes (DEGs) that would distinguish these groups and shed new light on the mechanisms of smoking-related carcinogenesis of the respiratory tract.

## Patients and Methods

### Study population

Participants in the study consisted of four nonsmokers (NS) as well as 16 smokers without cancer (SNC) and 14 smokers with diagnosed NSCLC (*N* = 10) or HNC (N = 4) (SC, considered as a single group) seen at the Centre Hospitalier de Luxembourg (CHL). Informed consents were received and the project was approved by the Centre National d'Ethique et de Recherche du Luxembourg. Bronchoscopic procedures and processing of the biopsies were carried out according to protocols approved by the CHL ethics committee. The participants provided detailed smoking and medical history information to a trained interviewer (Table 1).

**Table 1:** Characteristics of the study population.

Group	Patient	Number of biopsies	Age	Sex	Pky	Smoking status^1^	COPD	Histology	Grade	Stage
Nonsmokers	NS-1	2	34	M	0		No			
	NS-2	5	48	M	0		No			
	NS-3	4	46	M	0		No			
	NS-4	3	53	F	0		No			
Smokers without cancer	SNC-1	3	50	M	30	Current	No			
	SNC-2	3	49	M	55	Current	No			
	SNC-3	3	46	M	50	Current	No			
	SNC-4	2	52	M	52	Current	Yes			
	SNC-5	3	61	M	60	Current	No			
	SNC-6	3	50	M	40	Current	No			
	SNC-7	3	68	M	48	Current	Yes			
	SNC-8	3	49	M	30	Current	Yes			
	SNC-9	3	36	F	32	Current	No			
	SNC-10	3	48	F	28	Current	Yes			
	SNC-11	3	61	M	50	Current	No			
	SNC-12	3	40	M	15	Current	No			
	SNC-13	3	65	M	45	Current	Yes			
	SNC-14	4	58	M	38	Current	Yes			
	SNC-15	3	59	M	39	Current	No			
	SNC-16	3	61	M	50	Current	Yes			
Smokers with cancer	SC-1	3	63	M	80	Former (12)	Yes	NSCLC—squamous cell carcinoma	T2 N2 M0	IIIa
	SC-2	2	45	M	50	Current	Yes	HNC—squamous cell carcinoma	T2 N0 M0	Ib
	SC-3	2	73	M	27	Current	No	NSCLC—other type	T2 N0 M0	Ib
	SC-4	3	68	M	60	Current	Yes	NSCLC—adenosquamous carcinoma	T1 N0 M0	Ia
	SC-5	1	60	M	60	Current	Yes	NSCLC—squamous cell carcinoma	Tis N0 M0	Ia
	SC-6	3	60	M	60	Former (0.834)	No	NSCLC—squamous cell carcinoma	T4 N2 M0	IIIb
	SC-7	2	44	M	36	Current	No	NSCLC—adenocarcinoma	T1 N0 M0	Ia
	SC-8	3	57	M	37	Current	No	NSCLC—adenocarcinoma	T1 N0 M0	Ia
	SC-9	2	52	M	28	Current	No	NSCLC—other type	T2N0 M0	Ib
	SC-10	3	48	M	52	Current	No	HNC—squamous cell carcinoma	T4 N1 M0	IIIa
	SC-11	3	58	M	38	Current	Yes	HNC—squamous cell carcinoma	T2 N0 M0	Ib
	SC-12	2	53	M	30	Former (2)	Yes	HNC—squamous cell carcinoma	T4 N2 M0	IIIb
	SC-13	3	52	F	32	Current	Yes	NSCLC—squamous cell carcinoma	T3 N3 M1	IV
	SC-14	3	53	M	20	Current	Yes	NSCLC—adenocarcinoma	T1 N1 M1	IV

The percentage of males in the NS, SNC, and SC groups was 75%, 87.5%, and 92.9%, respectively, whereas the mean age for these groups was 45.25 (standard deviation (SD) 6.98), 53.31 (SD 8.67), and 56.14 (SD 8.00), respectively. The mean pack-years of smoking (pack-years; Pky) for the SNC and SC groups were 41.3 (SD 11.62) and 43.57 (SD 16.41), respectively, whereas the percentage of smokers with chronic obstructive pulmonary disease (COPD) in these groups was 43.75% and 51.14%, respectively. SNC, smokers without cancer; SC, single group; NSCLC, non-small cell lung cancer; HNC, head and neck cancer.

1 under (), years from smoking cessation to time of biopsy.

### Biopsy procedure

Bronchoscopic biopsies were taken either from NS volunteers, or during examination of smoking volunteers with or without suspicion of cancer. NSCLC and HNC diagnostics were confirmed by histopathology. Biopsies were taken during flexible video bronchoscopy under combined white-light and autofluorescence endoscopy (Karl Stortz endoscope). The bronchoscopic procedure lasted between 20 and 30 min. Patients were asked not to smoke the morning of the endoscopy. Biopsies from NSCLC patients were obtained from the contralateral lobe, tumor-distant sites in a homolateral lobe, or main carena (Fig. S1).

At each site, two adjacent biopsies were taken; one for RNA preparation, the other for histopathological analyses. The RNA of a given biopsy was used for gene profiling only when no cancerous or precancerous lesions were detected in the adjacent biopsy. To minimize random gene expression variations, three biopsies per individual were used for microarray analysis on average (Table 1).

### Sample preparation

Biopsy specimens (<20 *μ*m^3^) were immediately homogenized into 1 mL of TriPure isolation reagent (Roche Diagnostics, Mannhein, Germany), followed by RNA preparation as per the manufacturer's protocol. The RNAs were further purified by phenol–chloroform extraction and precipitation, and their integrity was verified using an Agilent 2100 BioAnalyzer (Agilent Technologies, Santa Clara, CA).

### Microarray data acquisition

Total RNAs (100 ng) were processed according to the Affymetrix GeneChip®3′IVT Express Kit User Manual (P/N 702646 Rev.1, High Wycombe, U.K.), and hybridized to Affymetrix HG-U133 Plus 2.0 array containing 54,675 probe sets representing over 47,000 human transcripts.

Microarray data are available in the ArrayExpress database (http://www.ebi.ac.uk/arrayexpress) under accession number E-MTAB-1690.

### Preprocessing of array

The Affymetrix CEL files were analyzed using a standard pipeline of Partek® Genomics Suite™. Preprocessing of raw data was carried out using the GCRMA method with quantile normalization 18. Standard quality metrics of Partek and principle component analysis (PCA) were used to detect potential outliers. Arrays were kept for further analysis based on the results of quality assessment (data not shown). Probe sets were further summarized to the gene level by averaging, resulting in 20,766 annotated genes. Distribution of the standardized gene log2 expressions were close to normal *z*-distribution (data not shown).

### Microarray data analysis and class prediction

Intraindividual variability was observed between biopsies, which was not consistently linked to the localization of the sites where biopsies had been obtained (data not shown). For these reasons, the 97 biopsies were treated individually for gene expression profiling. Statistical analysis was performed using R/Bioconductor. DEGs were detected using the empirical Bayes method provided by *limma* package 19. DEG lists were generated using filtering based on the false discovery rate (FDR) adjusted *P*-value (Benjamini–Hochberg correction) and the log-fold change (logFC) of gene expression. Detection of gene markers was performed using Tibshirani's prediction analysis for microarray (PAM) method, realized in *pamr* R package 20.

As indicated in Table 1, several smokers were affected by chronic obstructive pulmonary disease (COPD). COPD effect was addressed using the same statistical approach. We observed a small number of COPD-specific genes in the comparison of COPD-positive and COPD-negative SNC individuals (153 DEG with FDR < 0.01, of which none with |logFC|>1). At the same time, only three COPD-specific DEG were observed for SC individuals.

Multiclass LIMMA analysis of the SC group did not return any gene differentially expressed by site of biopsy (three sites considered: contralateral, nonadjacent homolateral, carena). Analysis of the same group by smoking status (current vs. ex-smokers) only returned 1 DEG (FDR < 0.01). No DEG was identified when current smokers with or without cancer were divided into two groups according to pack-years (Pky) status (Pky < 40 or Pky > 40) and subjected to 2-class analysis (FDR < 0.01).

Our collection of SC biopsies represents five cancer stages (Ia: *n* = 9; Ib: *n* = 9; IIIa: *n* = 6; IIIb: *n* = 5; and IV: *n* = 6) (Table 1). When gene expression profiles were compared through all stages, we identified a set of 47 genes that were significantly altered (FDR < 0.01). Principal component analysis and hierarchical clustering showed that the SC biopsies could be divided into five groups representing the five cancer stages based on expression of these genes (Fig. S2). However, due to the small number of biopsies available for each stage, this observation was not investigated further. It thus remains to be seen to what extent the observed changes in gene expression reflect stage-dependent systemic effects of the tumors.

### Functional classification of differentially expressed genes

Overrepresented functions and canonical pathways were identified using Ingenuity Pathway Analysis (IPA; Ingenuity Systems, Redwood City, CA; release date 08 November 2012). A pathway was considered to be significantly enriched when a score >2 (corresponding to a probability *P*-value <0.01) was found.

### Quantitative reverse transcription-polymerase chain reactions validation of selected microarray data

Reverse-transcriptase (RT) reactions were carried out using the Reverse Transcriptase Core kit (Eurogentec, Seraing, Belgium). Real-time polymerase chain reactions (PCR) analyses were performed on a 7300 Real-Time PCR System (Applied Biosystems, Life Technologies, Gent, Belgium), using the Power SYBR Green PCR master mix (Applied Biosystems) and the primers listed in Table S1. The housekeeping gene FLOT2 was used for normalization, and relative expression levels were calculated based on the cycle threshold (Ct) values, using the 2^−ΔΔCt^ method.

## Results

Thirty-four subjects, assigned to three groups (NS: *N* = 4; SNC: *N* = 16; and SC: *N* = 14, of which 10 NSCLC and four HNC, which were considered as a single group), were recruited for lung biopsy microarray analysis. RNAs from a total of 97 biopsies were used for gene expression profiling and analysis.

### Expression profile of cigarette smoking and NSCLC

We first carried out pairwise comparisons of the gene expression profiles obtained from NS, SNC, and SC in order to generate list of DEGs. Table S2 shows the numbers of DEGs obtained using the Bayesian method with various thresholds for the FDR and the expression fold change (FC). A large number of genes appeared to be differentially regulated in response to cigarette smoking, as illustrated by the two comparisons involving smokers and nonsmokers (SNC vs. NS, and SC vs. NS). In contrast, significantly less differential expression was observed in the comparison between SC and SNC. Therefore, for comparisons involving smokers and nonsmokers, we selected DEG list 1 (SNC vs. NS; 1359 genes) and DEG list 2 (SC vs. NS; 1391 genes) based on (FDR < 0.01) and (|logFC|>0.5). For the comparison between SC and SNC (DEG list 3; 416 genes), we omitted the logFC filter so that a significant number of genes could be considered for functional analyses. Note that in the following tables, the genes represented in a given list were selected solely on the base of the FDR, irrespective of the FC, so as to facilitate comparisons and discussion.

The top 50 DEGs of each list were selected for heatmap visualization (Fig. S3). Almost perfect separation of the SNC and NS groups was observed (panel A), and clustering of the SC and NS groups was faultless (panel B). Although clustering of the biopsies was not perfect in the case of SC versus SNC, correlated profiles were observed in most of the cases (panel C). Importantly, the major cluster of 25 SC biopsies identified in this analysis contained nine of the 10 HNC biopsies.

### Functional classification of the gene response to cigarette smoke and cancer of the respiratory tract

We next used IPA to analyze DEG lists 1–3. Pathways with an enrichment score >2 (corresponding to *P*-value <0.01) are listed in Table 2.

**Table 2:** Ingenuity Pathway Analysis (IPA) canonical pathways significantly associated with DEG lists 1–3.

DEG list 1 (SNC vs. NS)			DEG list 2 (SC vs. NS)		
Pathway	Score^1^	Ratio^2^	Pathway	Score	Ratio
Androgen and estrogen metabolism	2.35	0.167	Androgen and estrogen metabolism	2.84	0.182
Arachidonic acid metabolism	3.06	0.172	Glutathione metabolism	3.65	0.240
Glutamate metabolism	2.72	0.243	Glycosphingolipid biosynthesis—neolactoseries	2.12	0.261
Glycosphingolipid biosynthesis—neolactoseries	2.13	0.261	Metabolism of xenobiotics by cytochrome P450	3.53	0.184
Metabolism of xenobiotics by cytochrome P450	4.65	0.207	O-glycan biosynthesis	5.01	0.367
O-glycan biosynthesis	5.03	0.367	Pentose phosphate pathway	2.19	0.241
Pentose phosphate pathway	2.85	0.276	Retinol metabolism	3.31	0.237
Retinol metabolism	4.07	0.263			
Starch and sucrose metabolism	2.54	0.175			
Glutathione metabolism^3^	1.99	0.180			
DEG list 3 (SC vs. SNC)			DEG list 3 (SC vs. SNC) continued		
Pathway	Score	Ratio	Pathway	Score	Ratio
Allograft rejection signaling	8.19	0.169	Glycerolipid metabolism	3.06	0.088
Altered T-cell and B-cell signaling in rheumatoid arthritis	4.27	0.105	Glycolysis/gluconeogenesis	2.09	0.076
Antigen presentation pathway	5.12	0.175	Graft-versus-host disease signaling	7.95	0.217
Arachidonic acid metabolism	2.28	0.071	IL-17A signaling in airway cells	2.52	0.087
Autoimmune thyroid disease signaling	8.59	0.189	Metabolism of xenobiotics by cytochrome P450	6.83	0.138
B-cell development	2.52	0.138	NRF2-mediated oxidative stress response	4.96	0.080
Bile acid biosynthesis	4.07	0.146	Nur77 signaling in T lymphocytes	2.43	0.088
C21-steroid hormone metabolism	2.20	0.176	OX40 signaling pathway	2.43	0.082
Communication between innate and adaptive Immune cells	5.31	0.108	Pathogenesis of multiple sclerosis	3.05	0.333
Complement system	3.15	0.152	Primary immunodeficiency signaling	2.56	0.091
Crosstalk between dendritic cells and natural killer cells	2.56	0.078	Role of IL-17A in arthritis	2.87	0.100
Cytotoxic T lymphocyte-mediated apoptosis of target cells	4.47	0.135	Role of IL-17A in psoriasis	3.80	0.308
Dendritic cell maturation	3.40	0.064	Role of NFAT in regulation of the immune response	2.46	0.055
Differential regulation of cytokine production in intestinal epithelial cells by IL-17A and IL-17F	5.02	0.261	Role of pattern recognition receptors in recognition of bacteria and viruses	5.23	0.116
Differential regulation of cytokine production in macrophages and T helper cells by IL-17A and IL-17F	3.21	0.222	Systemic lupus erythematosus signaling	2.14	0.044
Fatty acid metabolism	2.18	0.069	TREM1 signaling	2.23	0.094
Glutathione metabolism	2.38	0.100	Type I diabetes mellitus signaling	5.48	0.105
			Xenobiotic metabolism signaling	3.31	0.058

NFAT, nuclear factor of activated T-cells.

1 IPA enrichment score (=−log *P*-value).

2 Ratio of genes represented in the gene list versus total genes in the pathway.

3 This pathway was included as it almost meets the selection criteria (score > 2) in DEG list 1.

We first analyzed the impact of cigarette smoke on the transcriptome by considering the comparisons between NS and smokers (SNC and SC) (DEG lists 1 and 2). A major pathway associated with these lists was Metabolism of xenobiotics by cytochrome P450. DEG lists 1 and 2 contained 13 xenobiotic biotransformation genes in common and, overall, gene expression followed an identical trend (up/downregulation) in these lists (Table 3).

**Table 3:** Genes belonging to the Metabolism of xenobiotic by Cyp450 pathway, identified in DEG lists 1–3.

Gene Symbol	Entrez gene name	DEG list 1 SNC vs. NS (logFC)^1^	DEG list 2 SC vs. NS (logFC)^1^	DEG list 3 SC vs. SNC (logFC)^1^
ADH1A	Alcohol dehydrogenase 1A (class I), *α* polypeptide		−0.477	−0.347
ADH1C	Alcohol dehydrogenase 1C (class I), *γ* polypeptide		−1.148	−0.975
ADH7	Alcohol dehydrogenase 7 (class IV), *μ* or *σ* polypeptide	1.474		−1.151
AKR1B10	Aldo-keto reductase family 1, member B10	4.365	2.491	−1.874
AKR1C1/AKR1C2	Aldo-keto reductase family 1, member C2	1.707	0.857	−0.943
AKR1C3	Aldo-keto reductase family 1, member C3	1.917	0.949	−0.968
AKR1C4	Aldo-keto reductase family 1, member C4	0.624		−0.338
ALDH3A1	Aldehyde dehydrogenase 3 family, member A1	2.712		−1.731
CSGALNACT1	Chondroitin sulfate *N*-acetylgalactosaminyltransferase 1	−1.035	−0.763	
CYP1A1	Cytochrome P450, family 1, subfamily A, polypeptide 1	3.957		
CYP1B1	Cytochrome P450, family 1, subfamily B, polypeptide 1	2.820	1.928	
CYP2A6	Cytochrome P450, family 2, subfamily A, polypeptide 6	−0.715	−0.620	
CYP2B6	Cytochrome P450, family 2, subfamily B, polypeptide 6			−0.701
CYP2C18	Cytochrome P450, family 2, subfamily C, polypeptide 18	1.016	0.618	
CYP3A5	Cytochrome P450, family 3, subfamily A, polypeptide 5	1.528		
CYP4F11	Cytochrome P450, family 4, subfamily F, polypeptide 11	0.879		
DHRS9	Dehydrogenase/reductase (SDR family) member 9	1.303	0.940	
GSTA1	Glutathione *S*-transferase *α* 1			−0.408
GSTA4	Glutathione *S*-transferase *α* 4			−0.351
GSTM1	Glutathione *S*-transferase *μ* 1	−0.449	−0.711	
GSTM2	Glutathione *S*-transferase *μ* 2 (muscle)	−0.459	−0.726	
GSTM3	Glutathione *S*-transferase *μ* 3 (brain)		−0.819	
GSTM5	Glutathione *S*-transferase *μ* 5	−0.847	−1.155	
GSTP1	Glutathione *S*-transferase pi 1	0.691		
GSTT1	Glutathione *S*-transferase *θ* 1	1.911	1.355	
GSTT2/GSTT2B	Glutathione *S*-transferase *θ* 2		−0.845	
MGST1	Microsomal glutathione *S*-transferase 1	0.469		−0.437
UGT1A6	UDP glucuronosyltransferase 1 family, polypeptide A6	0.902		−0.813
UGT1A9	UDP glucuronosyltransferase 1 family, polypeptide A9	0.667	0.573	
UGT2A1	UDP glucuronosyltransferase 2 family, polypeptide A1, complex locus		−1.400	

1 Log fold changes are given for all genes satisfying the selection criteria (FDR < 0.01).

Involvement of xenobiotic biotransformation enzymes in the formation and/or metabolism of several endogenous molecules (e.g., cholesterol, lipids, androgens, estrogens, and arachidonic acid metabolites) explained why the related canonical pathways were also enriched in DEG lists 1 and 2 (Table 2). Among the pathways also associated with these two lists was the O-Glycan Biosynthesis pathway involved in smoking-induced biosynthesis of mucin glycoproteins 21, and pathways containing smoking-induced antioxidant related genes involved in the pentose phosphate cycle and glutathione metabolism 22.

The impact of NSCLC on the transcriptome of smokers was then analyzed in DEG list 3. Metabolism of xenobiotic by cytochrome p450 was significantly enriched in DEG list 3 (Table 2); however, in contrast to DEG lists 1 and 2, the genes of DEG list 3 were all downregulated in SC compared with SNC (Table 3).

Also present in DEG list 3 were Xenobiotic metabolism signaling (15/261 genes represented) and NRF2-mediated oxidative stress response (15/187 genes represented), whose responses are crucial to limit oxidative damage. Strikingly, with the exception of IL1B, MAP2K6, and SOD2, which were upregulated, the other genes of these pathways were all downregulated in SC compared to SNC (Table S3).

Finally, more than 20 pathways associated with inflammatory processes and innate/adaptive immune responses were also enriched in DEG list 3, including several IL-17A-dependent pathways (Table 2). Table 4 shows the genes comprising these pathways with the changes in gene expression. With rare exceptions, these genes were all upregulated in SC compared to SNC.

**Table 4:** Genes comprising the various pathways associated with inflammatory processes and innate/adaptive immune response, in all 3 DEG lists.

Gene symbol	Entrez gene name	DEG list 1 SNC vs. NS (logFC)^1^	DEG list 2 SC vs. NS (logFC)^1^	DEG list 3 SC vs. SNC (logFC)^1^
ADA	Adenosine deaminase			0.409
C1QA	Complement component 1, q subcomponent, A chain			0.656
C1QB	Complement component 1, q subcomponent, B chain			0.855
CALM1	Calmodulin 1 (phosphorylase kinase, delta)			0.238
CCL20	Chemokine (C-C motif) ligand 20			0.982
CCL3	Chemokine (C-C motif) ligand 3			0.600
CCL4	Chemokine (C-C motif) ligand 4			0.648
CCR1	Chemokine (C-C motif) receptor 1			0.633
CD19	CD19 molecule			0.430
CD28	CD28 molecule			0.198
CD72	CD72 molecule			0.496
CD74	CD74 molecule, major histocompatibility complex, class II invariant chain			0.419
CD86	CD86 molecule			0.454
CFB	Complement factor B		1.176	1.248
CFI	Complement factor I			0.579
CR1	Complement component (3b/4b) receptor 1 (Knops blood group)		0.342	0.293
CXCL13	Chemokine (C-X-C motif) ligand 13			1.606
CXCL3	Chemokine (C-X-C motif) ligand 3			0.518
DDX58	DEAD (Asp-Glu-Ala-Asp) box polypeptide 58			0.386
DEFB4A/DEFB4B	Defensin, *β* 4A		0.568	0.377
EIF2AK2	Eukaryotic translation initiation factor 2-*α* kinase 2		0.328	0.198
FCER1A	Fc fragment of IgE, high-affinity I, receptor for; *α* polypeptide			−0.597
FCGR1A	Fc fragment of IgG, high-affinity Ia, receptor (CD64)		0.863	0.546
FCGR1B	Fc fragment of IgG, high-affinity Ib, receptor (CD64)		0.980	1.114
GNB4	Guanine nucleotide-binding protein (G protein), *β* polypeptide 4			0.336
GZMB	Granzyme B (granzyme 2, cytotoxic T-lymphocyte-associated serine esterase 1)			1.083
HLA-A	Major histocompatibility complex, class I, A		0.569	0.362
HLA-C	Major histocompatibility complex, class I, C			0.318
HLA-DMA	Major histocompatibility complex, class II, DM *α*			0.689
HLA-DMB	Major histocompatibility complex, class II, DM *β*			0.629
HLA-G	Major histocompatibility complex, class I, G		0.539	0.337
IFIH1	Interferon induced with helicase C domain 1		0.282	0.255
IL10	Interleukin 10		0.306	0.299
IL17RA	Interleukin 17 receptor A			0.234
IL1B	Interleukin 1, *β*			0.533
IRF7	Interferon regulatory factor 7			0.485
JAK3	Janus kinase 3			0.177
LCN2	Lipocalin 2		1.362	0.800
MAP2K6	Mitogen-activated protein kinase kinase 6		0.389	0.346
MAPKAPK2	Mitogen-activated protein kinase-activated protein kinase 2		0.257	0.146
MICB	MHC class I polypeptide-related sequence B			0.563
MMP13	Matrix metallopeptidase 13 (collagenase 3)			0.792
MUC5AC/MUC5B	Mucin 5AC, oligomeric mucus/gel-forming	1.910	1.148	−0.762
OAS2	2′-5′-oligoadenylate synthetase 2, 69/71 kDa		0.613	0.420
OAS3	2′-5′-oligoadenylate synthetase 3, 100 kDa		0.391	0.368
PLCB4	Phospholipase C, *β* 4			−0.410
PRF1	Perforin 1 (pore-forming protein)			0.429
RELB	v-rel reticuloendotheliosis viral oncogene homolog B			0.339
RFX5	Regulatory factor X, 5 (influences HLA class II expression)			0.218
SOCS1	Suppressor of cytokine signaling 1			0.139
TAP2	Transporter 2, ATP-binding cassette, sub-family B (MDR/TAP)			0.334
TLR2	Toll-like receptor 2		0.461	0.463

1 Log fold changes are given for all genes satisfying the selection criteria (FDR < 0.01).

Finally, when compared with a 240-probe-set signature found to be associated with NF-*κ*B in human lung carcinoma cell lines 23, DEG list 3 revealed an overlap of 15 NF-*κ*B-responsive genes (ABCC3, ALDH3A2, C3, CCL20, CXCL3, DHRS3, GCH1, HLA-C, IFIH1, PARP12, RELB, TAP1, TAP2, TLR2, CFB) involved in inflammation and adaptive/innate immunity.

### Gene signatures of cigarette smoking and cancer of the respiratory tract

The prediction method PAM was used to identify putative signatures that distinguished SNC and NS, SC and NS, and SC and SNC.

**Figure 1:**
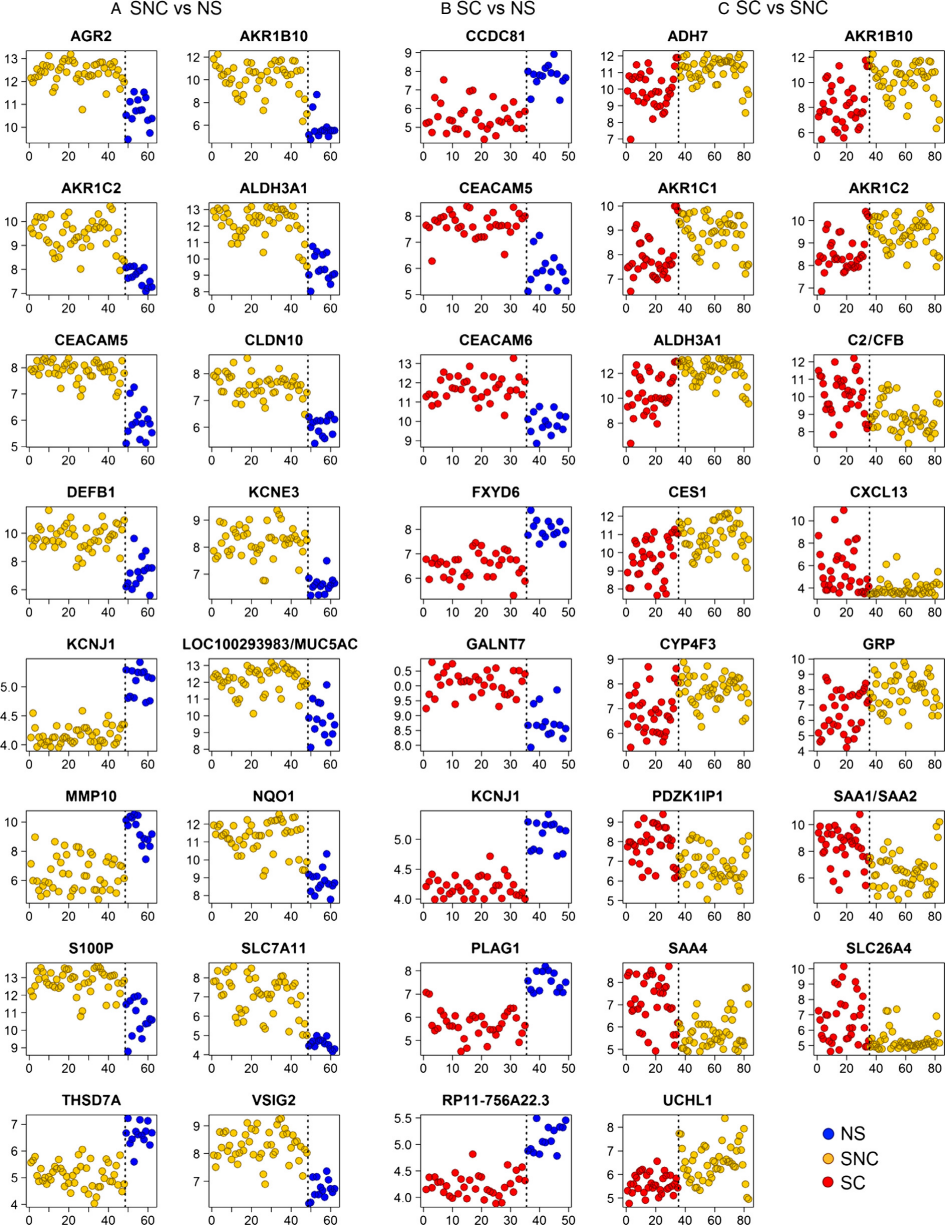
Expression values of the genes composing the signatures that distinguishes: (A) SNC and NS, (B) SC and NS, (C) SC and SNC. Shown are the log2 expression values (vertical axis) obtained from the microarray data for each biopsy (horizontal axis). Classes are separated by a dashed vertical line. SNC, smokers without cancer; NS, nonsmokers; SC, smokers with cancer.

A 16-gene signature was found that distinguished SNC and NS with an accuracy of 0.952. The genes comprising this signature are presented in Table 5, and their behavior in the SNC and NS groups, in Figure 1A. We evaluated this signature for its ability to distinguish between NS and SNC in two previously published microarray datasets: the airway epithelial gene expression studies of Spira et al. 9. and Ammous et al. 24. Our signature allowed clustering of the NS and SNC groups from these studies with an accuracy of 100% and 97.2%, respectively (Fig. S4).

**Table 5:** Gene signatures of cigarette smoking and cancer of the respiratory tract.

Gene symbol	Entrez gene name	logFC^2^	Adj. *P*-value^2^
SNC-vs-NS^1^			
AKR1B10	Aldo-keto reductase family 1, member B10	4.360	8.08E-13
ALDH3A1	Aldehyde dehydrogenase three family, member A1	2.710	8.19E-09
DEFB1	Defensin, *β* 1	2.500	2.38E-11
SLC7A11	Solute carrier family 7, (cationic amino acid transporter, *y* + system) member 11	2.480	2.83E-11
NQO1	NAD(P)H dehydrogenase, quinone 1	2.470	2.60E-10
S100P	S100 calcium-binding protein P	2.060	2.89E-11
CEACAM5	Carcinoembryonic antigen-related cell adhesion molecule 5	1.940	1.03E-20
AKR1C2	Aldo-keto reductase family 1, member C2	1.760	6.82E-11
AGR2	Anterior gradient homolog 2 (Xenopus laevis)	1.710	3.08E-11
KCNE3	Potassium voltage-gated channel, Isk-related family, member 3	1.590	1.09E-10
VSIG2	V-set and immunoglobulin domain containing 2	1.560	3.38E-10
CLDN10	Claudin 10	1.510	1.01E-14
MUC5AC	Mucin 5AC, oligomeric mucus/gel-forming	1.440	7.60E-09
KCNJ1	Potassium inwardly-rectifying channel, subfamily J, member 1	−0.912	1.45E-19
THSD7A	Thrombospondin, type I, domain containing 7A	−1.520	1.77E-12
MMP10	Matrix metallopeptidase 10 (stromelysin 2)	−2.930	7.82E-07
SC-vs-NS^1^			
CEACAM6	Carcinoembryonic antigen-related cell adhesion molecule 6 (nonspecific cross-reacting antigen)	1.780	9.61E-10
CEACAM5	Carcinoembryonic antigen-related cell adhesion molecule 7	1.740	1.33E-16
GALNT7	UDP-*N*-acetyl-*α*-d-galactosamine:polypeptide *N*-acetylgalactosaminyltransferase 7 (GalNAc-T7)	1.330	2.36E-12
KCNJ1	Potassium inwardly rectifying channel, subfamily J, member 1	−0.873	6.81E-17
RP11-756A22.3	Transmembrane phosphoinositide 3-phosphatase and tensin homolog two pseudogene	−0.879	2.88E-14
FXYD6	FXYD domain containing ion transport regulator 6	−1.450	2.55E-14
PLAG1	Pleiomorphic adenoma gene 1	−1.820	1.38E-10
CCDC81	Coiled-coil domain containing 81	−2.190	2.78E-10
SC-vs-SNC^1^			
SAA1///SAA2	Serum amyloid A1///serum amyloid A2	1.870	1.01E-05
CXCL13	Chemokine (C-X-C motif) ligand 13	1.610	7.71E-04
SAA4	Serum amyloid A4, constitutive	1.360	1.01E-05
SLC26A4	Solute carrier family 26, member 4	1.350	1.40E-04
C2///CFB	Complement component 2///complement factor B	1.250	3.79E-05
PDZK1IP1	PDZK1 interacting protein 1	1.020	2.54E-04
UCHL1	Ubiquitin carboxyl-terminal esterase L1 (ubiquitin thiolesterase)	−0.881	4.29E-06
CYP4F3	Cytochrome P450, family 4, subfamily F, polypeptide 3	−0.886	1.84E-04
AKR1C2	Aldo-keto reductase family 1, member C2	−0.901	1.04E-04
AKR1C1	Aldo-keto reductase family 1, member C1	−0.986	5.71E-05
ADH7	Alcohol dehydrogenase 7 (class IV), *μ* or *σ* polypeptide	−1.150	4.27E-04
CES1	Carboxylesterase 1 (monocyte/macrophage serine esterase 1)	−1.200	1.15E-04
GRP	Gastrin-releasing peptide	−1.330	8.71E-04
ALDH3A1	Aldehyde dehydrogenase three family, member A1	−1.730	3.68E-05
AKR1B10	Aldo-keto reductase family 1, member B10	−1.870	1.75E-04

1 Signatures providing optimal distinction in pairwise comparisons between the indicated groups are shown.

2 Given are log fold changes and adjusted *P*-values specific to the indicated comparison.

Another signature of eight genes distinguished SC and NS with an accuracy of 100% (Table 5 and Fig. 1B).

**Figure 2:**
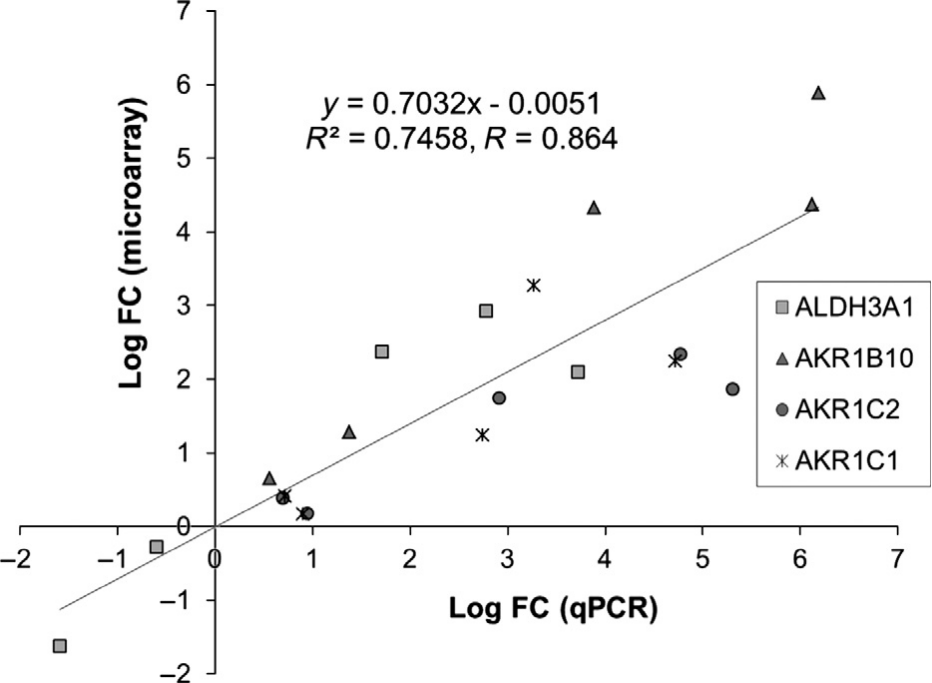
Validation of the expression data obtained for a subset of the genes composing the 15-gene signature that distinguishes between SC and SNC. Fold changes of expression (indicated as logFC) obtained by microarray analysis of the indicated genes in a selection of six biopsies were plotted against those obtained using reverse transcription-quantitative polymerase chain reaction (RT-qPCR) analysis, followed by correlation analysis. SNC, smokers without cancer; SC, smokers with cancer.

Importantly, a 15-gene signature discriminated SC and SNC with an accuracy of 83.2% (Table 5 and Fig. 1C). Four genes out of this signature (ALDH3A1, AKR1B10, AKR1C1, and AKR1C2) were technically validated by quantitative RT-PCR analysis on a subset of six biopsy specimens (one NS, three SNC, and two SC). Overall, a correlation coefficient of 0.864 was calculated from the plot shown in Figure 2, supporting the validity of our microarray analyses.

We found no overlap between our 15-gene signature and two lists of 55 and 21 genes, respectively, identified as commonly differentially expressed between normal lung tissue and adenocarcinomas or squamous cell carcinomas 25. Likewise, our signature did not contain any of the most frequently reported dysregulated genes in microarray studies comparing HN squamous cell carcinoma versus normal mucosa 26 (data not shown).

To gain more insight into the relevance of our 15-gene signature, we examined the presence of its genes in published lung and HN gene signatures (*n* = 49) of the GeneSigDB database 27. We identified a subset of 13 signatures in which our gene list was significantly overrepresented (*P*-value <0.05) (Fig. S5). We also identified three members of our signature (AKR1B10, AKR1C1, and AKR1C2) in the lists of genes dysregulated in NSCL from Woenckhaus et al. 15.

Gene expression profiling of cytologically normal epithelial cell brushings by Spira et al. 12 led to an 80-gene biomarker that distinguished between smokers with or without lung cancer with an accuracy of 83%. This signature, however, was not able to cluster our biopsies from smokers with and without cancer, even when we restricted the cancer group to NSCLC (i.e., omitting HNC biopsies). Likewise, our 15-gene signature failed to distinguish SNC and SC in the author's cohort (data not shown). As a step to understand the basis behind these observations, we directly compared the lists of DEGs obtained in this study for the comparisons of SNC versus NS, and SC versus SNC, with those we established from the corresponding datasets of Spira et al. For the comparison of SNC and NS, lists of the top 100 DEGs (FDR < 0.01) built from our data and those of Spira et al. 9 shared 19 genes in common (Table S4). In contrast, no genes were found in common between our list and that built from the data of Spira et al. 12 for the comparison of smokers with or without cancer. In addition, there was no overlap between the 80-gene biomarker of Spira et al. 12 and our 15-gene signature.

Analysis of our tumor-distant SC biopsies by site (homolateral, contralateral, carena) did not return any gene differentially expressed (data not shown). In addition, there was no significant overlap between our DEG list 3 or its derived 15-gene signature, and gene features found to be differentially expressed by site in a recently published gene expression study of bronchial airway epithelial cells in early-stage smoker NSCLC patients having undergone resective surgery 28. Likewise, neither DEG list 3 nor the 15-gene signature were significantly enriched in genes that constitute the published signature of the airway basal cells, the stem/progenitor cells of the human airway epithelium^29^,^30^ (data not shown). Finally, DEG list 3 did not contain any of the 26 testis-specific/placenta-specific genes recently found to be activated in lung tumors and associated with an aggressive phenotype^31^ (data not shown).

## Discussion

This study assessed the transcriptional profiling of histologically normal bronchial biopsy specimens obtained from NS as well as current smokers with or without NSCLC or HNC, in order to identify gene expression changes associated to cigarette smoking and smoking-related cancer of the respiratory tract.

For this study, we have considered NSCLC and HNC as a single group. Our heatmap analysis carried out with SC and SNC showed that HNC and NSCLC biopsies largely clustered together. Several studies have supported the notion that the field of tissue injury induced by cigarette smoke impacts the entire respiratory tract, including the oral and nasal mucosa 14. Our study of histologically normal bronchial biopsy specimens adds weight to this notion, suggesting that such biopsies are also informative of carcinogenic events affecting the upper aerodigestive tract.

Enrichment of the Metabolism of xenobiotics by cytochrome P450 pathway was observed in all 3 DEG lists. This pathway contains oxidoreductases involved in the detoxification of xenobiotics and their potential activation into genotoxic carcinogens and metabolic poisons. In agreement with previous studies 9,13,15,32,33, we observed the induction, in response to cigarette smoke, of several genes encoding xenobiotic biotransformation enzymes, including AKR1C1/C2, AKR1B10, CYP1A1, and CYP1B1, which play crucial roles in the metabolism/activation of polycyclic aromatic hydrocarbons, an important group of procarcinogens contained in tobacco smoke 5,34.

In contrast, downregulation was noted for the xenobiotic biotransformation genes of DEG list 3. With rare exceptions, we also observed the downregulation of most of the genes comprising the Xenobiotic metabolism signaling and NRF2-mediated oxidative stress response pathways, in SC compared to SNC. These observations suggest that key signaling and metabolic pathways contributing to the cellular response to cigarette smoke and oxidants are downregulated in the lungs of smokers with cancer. Downregulation of crucial antioxidant defense genes associated with lung cancer was previously noted in a gene expression profiling study of epithelial cell brushings 12.

Avoiding immune destruction and tumor-promoting inflammation are emerging hallmarks of cancer 35. Immune dysfunction is reported in lung cancer 36 and HNC 37. This study found evidence for the upregulation of important components of inflammation as well as innate/adaptive immune responses in the histologically normal mucosa of smokers with cancer of the respiratory tract.

Several IL-17A-dependent pathways were associated with DEG list 3. IL-17A cytokines, which are produced by T-helper (Th) 17 cells and also pulmonary macrophages and neutrophils 38, play an important role in the pathogenesis of respiratory disease 39. The immune response orchestrated by Th17 cells is linked to the chemokine/chemokine receptor pair CCL20/CCR6 which is involved in smoke-related chronic inflammatory pathologies 40,41. Activation of the axis composed of CCL20/CCR6 and IL-17 is involved in NSCLC progression, and elevated intratumoral levels of IL-17RA and CCL20 proteins have been observed, as compared to tumor-adjacent lung tissue 42. Th17 cells also actively migrate to the tumor milieu and have been shown to exert a substantial impact on the carcinogenesis of HNC 43. Our data suggest that activation of the CCL20/CCR6/IL-17 axis is also detectable in the histologically normal mucosa of smokers with cancer of the respiratory tract.

HLA-G, involved in the suppression of innate/adaptive immune response in lung cancer 36, and the immunosuppressive cytokine IL-10 44 were upregulated in SC compared to SNC, suggesting that signs of tumor immune evasion mechanisms can be detected in the tumor-distant, histologically normal mucosa of smokers with cancer.

The observed upregulation of RELB might reflect the activation of an alternative NF-*κ*B pathway to limit inflammation 45,46, whereas upregulation of the TLR family of pattern-recognition receptors, TLR2, may indicate chronic inflammation or tumor immune escape mechanisms following exposure to damage-associated molecular patterns (DAMPs) released from the injured tissue/tumor 47. Interestingly, one DAMP recognized by TLR2 is serum amyloid A (SAA) 48, which we found upregulated in patients with NSCLC (Table 3). Whether upregulation of TLR2 reflects an exacerbation of the inflammatory response in these patients remains to be elucidated.

This study has also led to three gene signatures that distinguish between NS, SNC, and SC. Our 16-gene signature distinguishing SNC and NS, could also separate these groups of patients in two published datasets obtained from transcriptomic analyses of epithelial cell brushings. Histologically normal biopsy specimens and epithelial brushings thus appear to provide compatible information on alterations of the transcriptome when considering the impact of cigarette smoke on the field of tissue injury in the lung and airway of healthy individuals.

A 15-gene signature distinguished between SNC and SC with an accuracy of 83%. The limited number of patients in our study precluded the assignment of training and control sets. Thus, further work will be necessary to test the potential of our signature as a biomarker of cancer of the respiratory tract in smokers. However, it is notable that a large number of these 15 genes are associated to smoking-related pathogenesis and/or carcinogenesis of the respiratory tract. The expression patterns and involvement of the xenobiotic metabolism/detoxification genes comprising the signature in these processes are well-documented (AKR1B10 15,49–52, AKR1C1/C2 15,49, ALDH3A1 49,53, CYP4F3 49, ADH7 54, CES1^55^). Whether the downregulation of these xenobiotic biotransformation genes in the histologically normal lung mucosa of SC, compared to SNC, reflects a shift in the balance between a protective role against the chemicals of cigarette smoke, and enzymatic activities that generate DNA-reactive metabolites and contribute to carcinogenesis 34 remains to be investigated.

Other relevant genes in our signature include pendrin/SLC26A4, a critical mediator for the production of mucus in bronchial asthma and COPD 56. In addition, increased SAA levels were detected in the serum of lung cancer patients and proposed as a prognostic lung cancer biomarker 57. Finally, UCHL1/PGP9.5, a proposed marker for NSCLC 58, is an oncogene that initiates the development of lung adenomas and adenocarcinomas in mice 59 and also functions as a tumor suppressor in HNC 60.

Several reviews have pointed to the limited gene overlap found between gene signatures previously identified in different gene expression profiling studies of tumors of the respiratory tract 10,25,26,61. Although our 15-gene signature did not contain any of the most frequently reported dysregulated genes in microarray studies of NSCLC 25 and HNC 26, we identified a subset of lung and HN gene signatures showing statistically significant gene overlap with our signature, including NSCLC and HNC signatures derived from gene expression profiling of dissected tumors and adjacent normal tissues. Thus, it is tempting to speculate that genes like AKR1B10, AKR1C1/C2, ALDH3A1, ADH7, CES1, CFB, GRP, PDKZ1P1, SAA, and UCHL1, which are found dysregulated both in the histologically normal bronchial mucosa and in resected NSCLC or HNC tumor samples, identify early events in smoking-associated carcinogenesis of the respiratory tract.

Although most published lung cancer gene signatures have been derived from gene expression profiling of resected tumors and adjacent normal tissues 10,25,62, recent studies from Spira et al. 12 and Kadara et al. 28 have assessed the molecular field of lung injury through gene expression profiling of cytologically normal epithelial cell brushings. However, neither Spira et al.'s 80-gene biomarker nor our 15-gene signature clustered SNC and SC correctly when challenged with each other's cohort, even when HNC biopsies were removed from our cohort. In addition, there was no gene overlap between lists of the top 100 DEGs established from ours and the author's dataset. One potential explanation is that the cancer patient cohort used by Spira et al. included not only NSCLC but also small cell lung cancer and unclassified cancer types. Another possibility is that inflammatory cells and other components of the stroma present in our biopsies, but not in the epithelial brushings studied by Spira et al., contribute importantly to our signature. This latter possibility could also contribute in part to the lack of overlap between our gene lists and that of Kadara et al. 28, although it is more likely that the lack of overlap with lists generated in this latter study stems from the fact that the author's study, unlike ours, considered comparisons involving samples adjacent to the tumor site.

The study of Kadara et al. 28 recently highlighted the importance of extending the exploration of the molecular field of lung injury beyond the tumor itself and its margins. In line with this study, our data support further exploration of tumor-distant, histologically normal bronchial biopsies to investigate the molecular mechanisms underlying smoking-related carcinogenesis of the respiratory tract.

## Supporting Information

**Figure S1.** Scheme illustrating the sites where benign lung biopsies were taken for the analysis of the smokingrelated molecular field of injury of the lung and airway in patients with NSCLC. See text for additional details. **Figure S2.** Genes differentially expressed amongst the five cancer stages of the SC biopsies. (A) Three-dimensional representation of the principal component analysis showing segregation of the 37 SC biopsies based on the 47 DEGs. (B) Hierarchical clustering of SC biopsies based on the 47 DEGs. See text for details. **Figure S3.** Hierarchical clustering and heatmap representation of the top 50 genes differentially expressed in the indicated comparisons of SNC versus NS, SC versus NS and SC versus SNC. The color codes for the groups and the color-coded scale (blue=downregulation, red=upregulation) for the standardized expression values are indicated, respectively, at the top and the bottom of the figure. HNC biopsies on the rightmost heatmap are indicated by bars. **Figure S4.** Validation of the 16-gene signature that distinguishes SNC and NS, on two independent datasets. Heatmaps for the signature on the datasets of (A) Spira et al. [9] and (B) Ammous et al. [24]. See text for details. **Figure S5.** (A) The 13 published lung and head and neck gene signatures displaying a significant enrichment in the genes composing the 15-gene signature that distinguishes between SC and SNC. Analysis was carried out using the GeneSigDB interface: Results of the enrichment test were filtered based on the corrected P-value (<0.05), gene overlap >1 (number of genes shared by each signature and the signature identified in this study) and relevance to lung and head and neck. The size of each gene-signature is indicated, as well as the gene overlap. Note that the numbers in the gene signature column refer to the PubMed index of each reference. (B). Heatmap representation of the presence (red square) or absence (gray square) of each gene composing the signature identified in this study, within the 18 gene-signatures identified in (A). See text for discussion.

Table S1 Primers and annealing temperatures used in real-time RT-PCR.

Table S2 Numbers of DEGs obtained with various thresholds for the false discovery rate and the expression fold change.

Table S3 Genes comprising the Xenobiotic metabolism signaling and NRF2-mediated oxidative stress response canonical pathways identified in DEG list 3, and their fold change of expression in all three lists.

Table S4 Gene overlap found between lists of the top 100 DEG for the comparison between SNC and SC, established for this study and that of Spira et al.
