# Supplementary material for: Bronchial airway gene expression in smokers with lung or head and neck cancer
Source: Cancer Med. 2014 Feb 4;3(2):322–36. doi: 10.1002/cam4.190 (PMC3987082; doi:10.1002/cam4.190)
Supplement: Supplementary file 1 — Figure S1. Scheme illustrating the sites where benign lung biopsies were taken for the analysis of the smokingrelated molecular field of injury of the lung and airway in patients with NSCLC. See text for additional details. Figure S2. Genes differentially expressed amongst the five cancer stages of the SC biopsies. (A) Three-dimensional representation of the principal component analysis showing segregation of the 37 SC biopsies based on the 47 DEGs. (B) Hierarchical clustering of SC biopsies based on the 47 DEGs. See text for details. Figure S3. Hierarchical clustering and heatmap representation of the top 50 genes differentially expressed in the indicated comparisons of SNC versus NS, SC versus NS and SC versus SNC. The color codes for the groups and the color-coded scale (blue=downregulation, red=upregulation) for the standardized expression values are indicated, respectively, at the top and the bottom of the figure. HNC biopsies on the rightmost heatmap are indicated by bars. Figure S4. Validation of the 16-gene signature that distinguishes SNC and NS, on two independent datasets. Heatmaps for the signature on the datasets of (A) Spira et al. [9] and (B) Ammous et al. [24]. See text for details. Figure S5. (A) The 13 published lung and head and neck gene signatures displaying a significant enrichment in the genes composing the 15-gene signature that distinguishes between SC and SNC. Analysis was carried out using the GeneSigDB interface: Results of the enrichment test were filtered based on the corrected P-value (<0.05), gene overlap >1 (number of genes shared by each signature and the signature identified in this study) and relevance to lung and head and neck. The size of each gene-signature is indicated, as well as the gene overlap. Note that the numbers in the gene signature column refer to the PubMed index of each reference. (B). Heatmap representation of the presence (red square) or absence (gray square) of each gene composing the signature identified in thi [file cam40003-0322-sd1.pdf]

Supplementary Figures
Van Dyck et al.

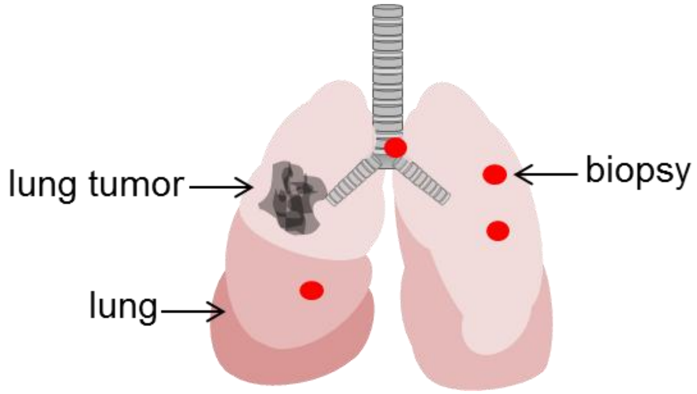

Figure S1. Scheme illustrating the sites where benign lung biopsies were taken for the analysis of the smoking-related molecular field of injury of the lung and airway in patients with NSCLC. See text for additional details.

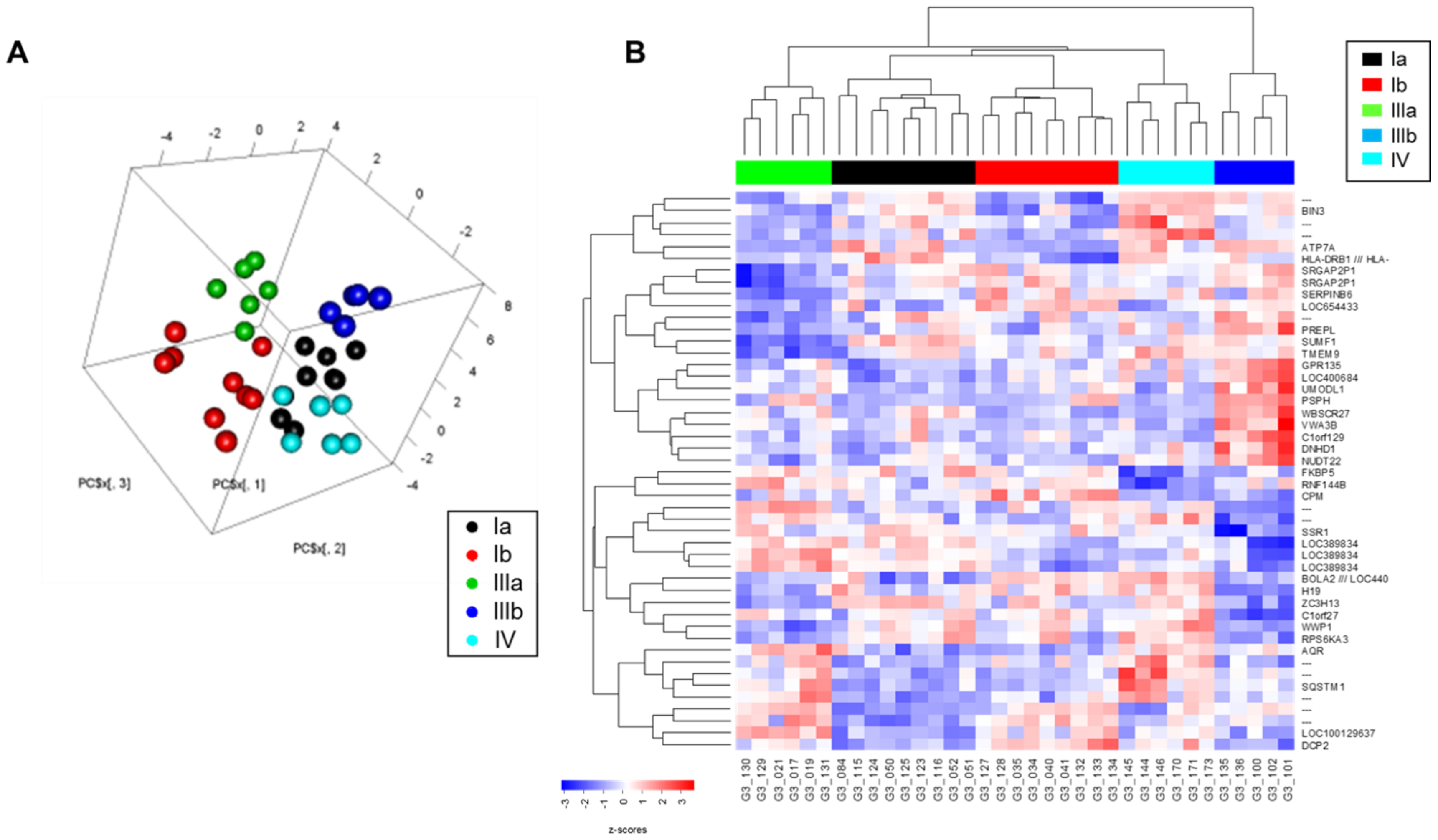

Figure S2. Genes differentially expressed amongst the 5 cancer stages of the SC biopsies. (**A**) 3-dimensional representation of the principal component analysis showing segregation of the 37 SC biopsies based on the 47 DEGs. (**B**) Hierarchical clustering of SC biopsies based on the 47 DEGs. See text for details.

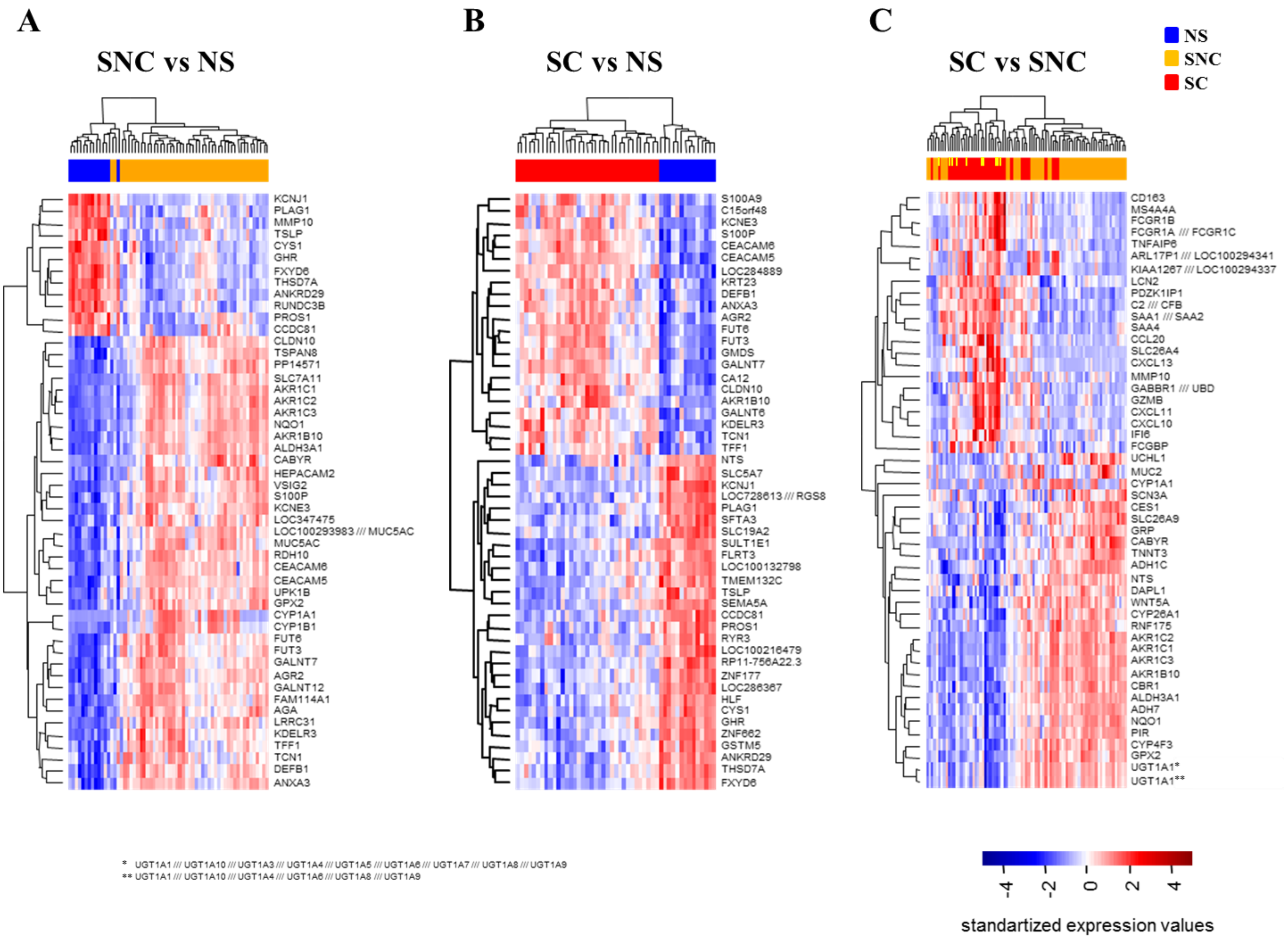
A
SNC vs NS
KCNJ1
PLAG1
MMP10
TSLP
CYS1
GHR
FXYD6
THSD7A
ANKRD29
RUNDC3B
PROS1
CCDC81
CLDN10
TSPAN8
PP14571
SLC7A11
AKR1C1
AKR1C2
AKR1C3
NQO1
AKR1B10
ALDH3A1
CABYR
HEPACAM2
VSIG2
S100P
KCNE3
LOC347475
LOC100293983 /// MUC5AC
MUC5AC
RDH10
CEACAM6
CEACAM5
UPK1B
GPX2
CYP1A1
CYP1B1
FUT6
FUT3
GALNT7
AGR2
GALNT12
FAM114A1
AGA
LRRC31
KDELR3
TFF1
TCN1
DEFB1
ANXA3
B
SC vs NS
S100A9
C15orf48
KCNE3
S100P
CEACAM6
CEACAM5
LOC284889
KRT23
DEFB1
ANXA3
AGR2
FUT6
FUT3
GMDS
GALNT7
CA12
CLDN10
AKR1B10
GALNT6
KDELR3
TCN1
TFF1
NTS
SLC5A7
KCNJ1
LOC728613 /// RGS8
PLAG1
SFTA3
SLC19A2
SULT1E1
FLRT3
LOC100132798
TMEM132C
TSLP
SEMA5A
CCDC81
PROS1
RYR3
LOC100216479
RP11-756A22.3
ZNF177
LOC286367
HLF
CYS1
GHR
ZNF662
GSTM5
ANKRD29
THSD7A
FXYD6
C
SC vs SNC
NS
SNC
SC
CD163
MS4A4A
FCGR1B
FCGR1A /// FCGR1C
TNFAIP6
ARL17P1 /// LOC100294341
KIAA1267 /// LOC100294337
LCN2
PDZK1IP1
C2 /// CFB
SAA1 /// SAA2
SAA4
CCL20
SLC26A4
CXCL13
MMP10
GABBR1 /// UBD
GZMB
CXCL11
CXCL10
IFI6
FCGBP
UCHL1
MUC2
CYP1A1
SCN3A
CES1
SLC26A9
GRP
CABYR
TNNT3
ADH1C
NTS
DAPL1
WNT5A
CYP26A1
RNF175
AKR1C2
AKR1C1
AKR1C3
AKR1B10
CBR1
ALDH3A1
ADH7
NQO1
PIR
CYP4F3
GPX2
UGT1A1*
UGT1A1**
* UGT1A1 /// UGT1A10 /// UGT1A3 /// UGT1A4 /// UGT1A5 /// UGT1A6 /// UGT1A7 /// UGT1A8 /// UGT1A9
** UGT1A1 /// UGT1A10 /// UGT1A4 /// UGT1A6 /// UGT1A8 /// UGT1A9
-4
-2
0
2
4
standartized expression values

Figure S3. Hierarchical clustering and heatmap representation of the top 50 genes differentially expressed in the indicated comparisons of SNC vs NS, SC vs NS and SC vs SNC. The color codes for the groups and the color-coded scale (blue = downregulation, red=upregulation) for the standardised expression values are indicated, respectively, at the top and the bottom of the figure. HNC biopsies on the rightmost heatmap are indicated by bars.

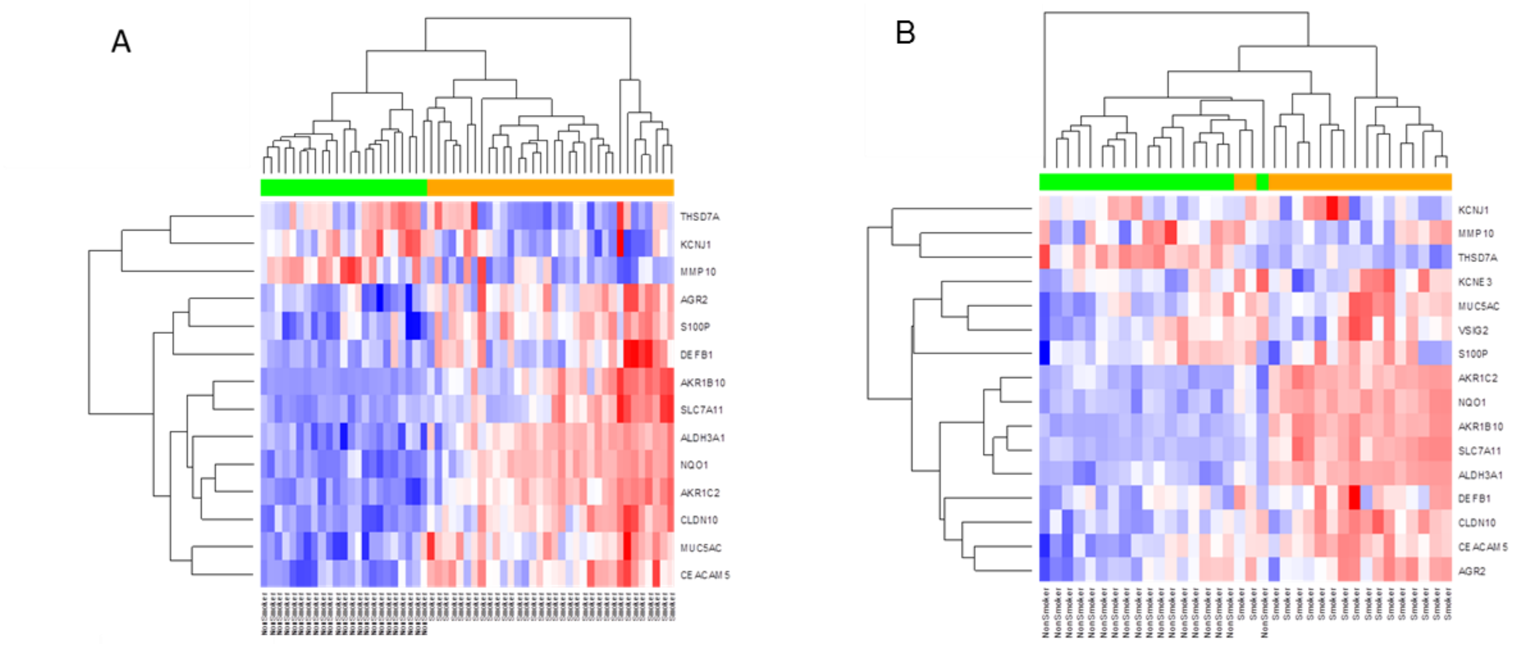

Figure S4. Validation of the 16-gene signature that distinguishes SNC and NS, on two independent datasets. Heatmaps for the signature on the datasets of (**A**) Spira et al. [10] and (**B**) Amous et al. [28]. See text for details.

A

Gene Signature*	Signature Name	Description	Reference	Size	Overlap	P-value
17115125-SuppTable2	Lung_not_cancer_Harvey06_152genes	Genes differentially expressed in the small airway epithelium of 6 healthy smokers vs 5 healthy non-smokers	1	125	9	0
17115125-SuppTable4	Lung_not_cancer_Harvey06_118genes	Genes differentially expressed in the small airway epithelium of 10 healthy smokers vs 12 healthy non-smokers	1	91	7	0
16912175-SuppTable3b	HeadandNeck_Sengupta06_429genes	Genes whose expression is down-regulated in nasopharyngeal carcinoma tumors vs normal tissues	2	356	4	0
19334046-TableS3a	Lung_Boelens09_111genes	Up-regulated genes in normal bronchial epithelium as compared to ex-smokers	3	97	4	0
20332630-Table4	Lung_not_cancer_Crul10_25genes_up	Top 25 of the most highly up-regulated genes between COPD patients with acute exacerbation vs stable COPD patients	4	23	3	0
15735009-Table1	Lung_Mahadevan05_18genes	Altered gene expression patterns in MCF-7 cells induced by the urban dust particulate complex mixture standard reference material 1649a.	5	18	2	0
17108109-Table2a	Lung_not_cancer_Carolan06_11genes	Microarray assessment of neuroendocrine cell-specific genes in normal nonsmokers normal smokers, smokers with early COPD and smokers with established COPD	6	10	2	0
14676830-Table3	HeadandNeck_Cromer04_55genes	Sequences decreased in tumours of the hypopharynx (55 genes, 12 of which were decreased sequences in common with previous studies)	7	51	2	0.0001
20421987-TableS5	Lung_Hou10_75genes	NSCLC histology signature (short)	8	58	2	0.0001
20035825-TableS7a	Lung_Mikkonen10_416genes	Up-regulated genes in A549 cells treated with 1 μM Dexamethasone or vehicle for 24h.	9	325	3	0.0003
20421987-TableS4	Lung_Hou10_518genes	NSCLC histology signature (long)	8	404	3	0.0006
16715129-SuppTable5	HeadandNeck_Jarvinen06_223genes	Statistically identified genes whose expression was decreased due to copy number losses in the primary tumors.	10	213	2	0.0031
16489042-SuppTable1a	HeadandNeck_Roepman06_396genes	Predictive genes showing negative correlation with lymph node metastasis	11	318	2	0.0109

B

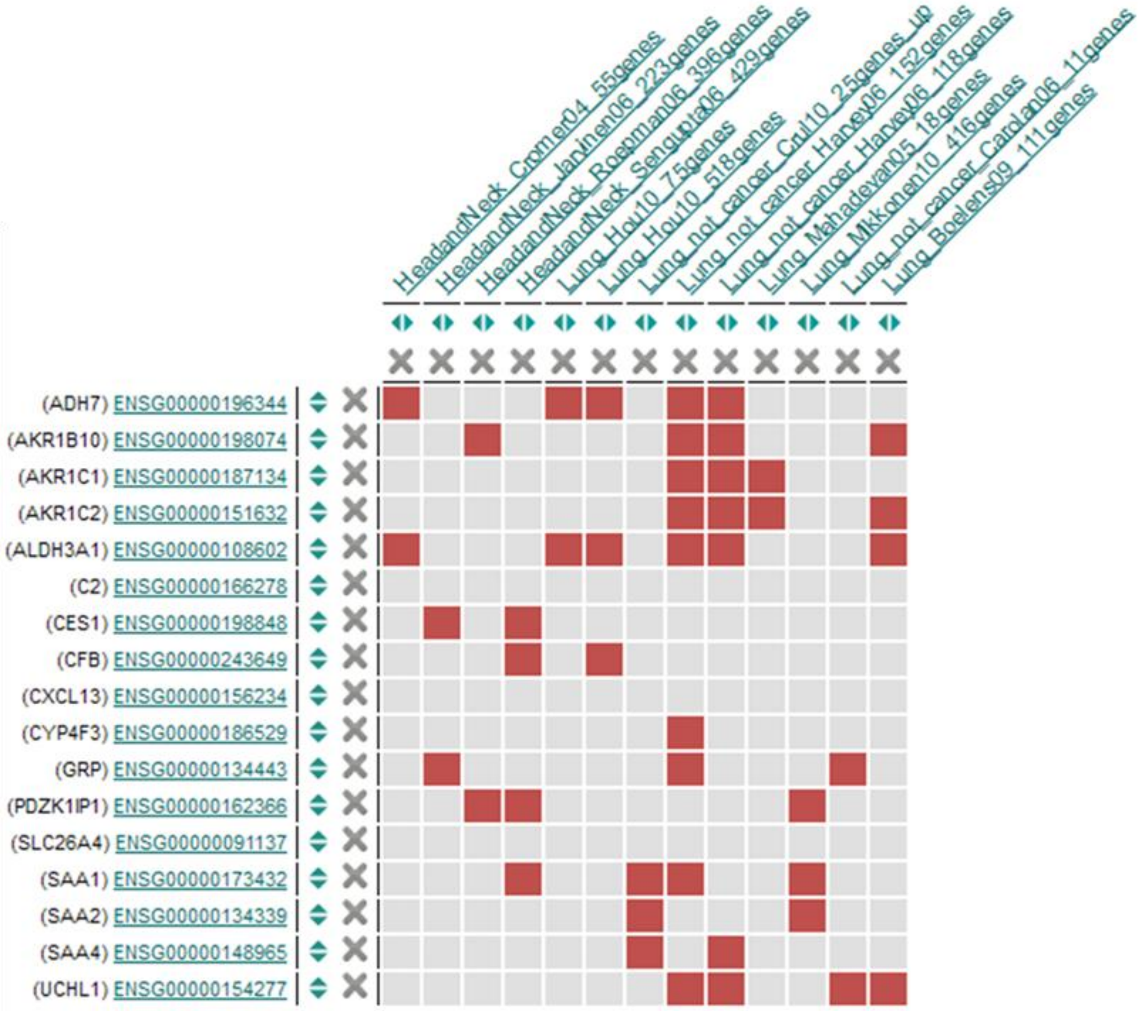
HeadandNeck_Cromer04_55genes
HeadandNeck_Jarvinen06_223genes
HeadandNeck_Roepman06_396genes
HeadandNeck_Sengupta06_429genes
Lung_Hou10_75genes
Lung_Hou10_518genes
Lung_not_cancer_Crul10_25genes_up
Lung_not_cancer_Harvey06_152genes
Lung_not_cancer_Harvey06_118genes
Lung_Mahadevan05_18genes
Lung_Mikkonen10_416genes
Lung_not_cancer_Carolan06_11genes
Lung_Boelens09_111genes
(ADH7) ENSG00000196344
(AKR1B10) ENSG00000198074
(AKR1C1) ENSG00000187134
(AKR1C2) ENSG00000151632
(ALDH3A1) ENSG00000108602
(C2) ENSG00000166278
(CES1) ENSG00000198848
(CFB) ENSG00000243649
(CXCL13) ENSG00000156234
(CYP4F3) ENSG00000186529
(GRP) ENSG00000134443
(PDZK1IP1) ENSG00000162366
(SLC26A4) ENSG00000091137
(SAA1) ENSG00000173432
(SAA2) ENSG00000134339
(SAA4) ENSG00000148965
(UCHL1) ENSG00000154277

Figure S5. (**A**) The 13 published lung and head and neck gene-signatures displaying a significant enrichment in the genes composing the 15-gene signature that distinguishes between SC and SNC. Analysis was carried out using the GeneSigDB interface: Results of the enrichment test were filtered based on the corrected P-value (<0.05), gene overlap >1 (number of genes shared by each signature and the signature identified in this study) and relevance to lung and head and neck. The size of each gene-signature is indicated, as well as the gene overlap. Note that the numbers in the gene-signature column refer to the PubMed index of each reference. (**B**). Heatmap representation of the presence (red square) or absence (grey square) of each gene composing the signature identified in this study, within the 18 gene-signatures identified in (A). See text for discussion.

References:

1. Harvey BG, Heguy A, Leopold PL, Carolan BJ, Ferris B, Crystal RG. Modification of gene expression of the small airway epithelium in response to cigarette smoking. Journal of molecular medicine 2007;85: 39-53.
2. Sengupta S, den Boon JA, Chen IH, Newton MA, Dahl DB, Chen M, Cheng YJ, Westra WH, Chen CJ, Hildesheim A, Sugden B, Ahlquist P. Genome-wide expression profiling reveals EBV-associated inhibition of MHC class I expression in nasopharyngeal carcinoma. Cancer Res 2006;66: 7999-8006.
3. Boelens MC, van den Berg A, Fehrmann RS, Geerlings M, de Jong WK, te Meerman GJ, Sietsma H, Timens W, Postma DS, Groen HJ. Current smoking-specific gene expression signature in normal bronchial epithelium is enhanced in squamous cell lung cancer. J Pathol 2009;218: 182-91.
4. Crul T, Testelmans D, Spruit MA, Troosters T, Gosselink R, Geeraerts I, Decramer M, Gayan-Ramirez G. Gene expression profiling in vastus lateralis muscle during an acute exacerbation of COPD. Cellular physiology and biochemistry : international journal of experimental cellular physiology, biochemistry, and pharmacology 2010;25: 491-500.
5. Mahadevan B, Keshava C, Musafia-Jeknic T, Pecaj A, Weston A, Baird WM. Altered gene expression patterns in MCF-7 cells induced by the urban dust particulate complex mixture standard reference material 1649a. Cancer Res 2005;65: 1251-8.
6. Carolan BJ, Heguy A, Harvey BG, Leopold PL, Ferris B, Crystal RG. Up-regulation of expression of the ubiquitin carboxyl-terminal hydrolase L1 gene in human airway epithelium of cigarette smokers. Cancer Res 2006;66: 10729-40.
7. Cromer A, Carles A, Millon R, Ganguli G, Chalmel F, Lemaire F, Young J, Dembele D, Thibault C, Muller D, Poch O, Abecassis J, et al. Identification of genes associated with tumorigenesis and metastatic potential of hypopharyngeal cancer by microarray analysis. Oncogene 2004;23: 2484-98.
8. Hou J, Aerts J, den Hamer B, van Ijcken W, den Bakker M, Riegman P, van der Leest C, van der Spek P, Foekens JA, Hoogsteden HC, Grosveld F, Philipsen S. Gene expression-based classification of non-small cell lung carcinomas and survival prediction. PLoS One 2010;5: e10312.
9. Mikkonen L, Pihlajamaa P, Sahu B, Zhang FP, Janne OA. Androgen receptor and androgen-dependent gene expression in lung. Molecular and cellular endocrinology 2010;317: 14-24.
10. Jarvinen AK, Autio R, Haapa-Paananen S, Wolf M, Saarela M, Grenman R, Leivo I, Kallioniemi O, Makitie AA, Monni O. Identification of target genes in laryngeal squamous cell carcinoma by high-resolution copy number and gene expression microarray analyses. Oncogene 2006;25: 6997-7008.
11. Roepman P, Kemmeren P, Wessels LF, Slootweg PJ, Holstege FC. Multiple robust signatures for detecting lymph node metastasis in head and neck cancer. Cancer Res 2006;66: 2361-6.
